# Analysis of loco-regional and distant recurrences in breast cancer after conservative surgery

**DOI:** 10.1186/s12957-016-0881-x

**Published:** 2016-05-14

**Authors:** Mostafa Elsayed, Mahmoud Alhussini, Ahmed Basha, A. T. Awad

**Affiliations:** General Surgery and Surgical Oncology, Alexandria University Students Hospital, Alexandria, Egypt; General Surgery and Surgical Oncology, Faculty of Medicine, Alexandria University, Alexandria, Egypt; General Surgery and Surgical Oncology, Alexandria, Egypt; Surgery Department, Faculty of Medicine, Alexandria University, Alexandria, Egypt

## Abstract

**Background:**

A number of patients treated conservatively for breast cancer will develop loco-regional and distant recurrences. Our aim was to determine how their occurrence may be linked to the evolution of the disease.

**Methods:**

We analyzed 238 women treated by conservative breast surgery and breast irradiation in a single institution. We evaluated the prognostic factors associated with loco-regional and distant recurrences and the prognostic value of local and regional recurrences on systemic progression.

**Results:**

After a median follow-up of 5 year (range 1–10), 16 (6.72 %) patients in the breast conservative surgery (BCS) groups had loco-regional recurrence. For distant recurrence, 10 (4.2 %) patients had experienced distant recurrence. Lympho-vascular invasion (HR 2.55; 95 % CI, 076 to 8.49) and an extensive intraductal component (HR, 2.22; 95 % CI, 0.69 to 7.15) and nodal status are risk factors for loco-regional recurrence (LRR) after breast conservative therapy (BCT). Tumor size, nodal status, high histologic grade, and breast cancer diagnosed at a young age (≤35 years) are correlated with higher distant recurrence rates after BCT.

**Conclusions:**

Risk factors for LRR after BCS include lympho-vascular invasion, extensive inraductal component, and high nodal status, where as risk factors for distant recurrence include tumor size, nodal status, high histologic grade, and breast cancer diagnosed at a young age (≤35 years).

## Background

The surgical treatment of breast cancer changed substantially over the past decades. There was a shift from the orthodox treatment applying modified radical mastectomy (MRM) to breast conservative surgery (BCS) with radiotherapy [1–4]; BCS became the standard treatment for patients with early breast cancer. It provides a better quality of life for these patients and has the same overall survival if compared with mastectomy. However, it is associated with a higher incidence of loco-regional recurrence. This event may be a biomarker of disease aggressiveness as distant spread is a frequent accompaniment [5–11].

In this study, we collected data on 238 women treated by BCS and breast radiotherapy in order to identify and assess the risk factors that might predict the occurrence of loco-regional and distant recurrence after BCS aiming at selecting the suitable patients with high risk of local breast recurrence after BCS.

## Methods

The data of operable patients, admitted and managed at the Surgical Oncology unit, Alexandria Faculty of Medicine, between 2005 and 2014, were retrospectively reviewed and analyzed.

The data of 238 patients were included; the following were recorded:

- An ethical approval statement was taken from all cases.
- Age at diagnosis: young patients are defined as younger than 35 years.
- Tumor characteristics: size, nodal status, presence of lympho-vascular invasion, amount of intraductal component, tumor grade, margin status, hormone receptor, and Her2 neu status.
- The follow-up period of the patients was registered.
- The occurrence of loco-regional recurrence or distant metastases during the follow-up period was recorded and considered as an end point for follow-up.

Local recurrence is defined as recurrence in the original tumor bed (for BCS) or field of mastectomy.

Regional recurrence refers to metastatic disease in the ipsilateral axilla or supraclavicular lymph nodes alone or in combination with the involvement of ipsilateral breast.

Loco-regional recurrence-free survival of patients who underwent BCS was estimated using the Kaplan-Meier method and compared among different categories using log-rank tests (univariable analysis of risk factors for loco-regional recurrence).

Distal recurrence-free survival for both groups will be analyzed using the same test (univariable analysis of risk factors for distal recurrence).

Associations with local recurrence after BCS were further evaluated using multivariable Cox proportional hazards regression model and summarized with hazard ratios 95 % confidence intervals (CIs).

Associations with distal recurrence after BCS were further evaluated using multivariable Cox regression model.

## Results

Age of 35 years or younger represented 21 (8.8 %) patients in this study. Two hundred seventeen (91.2 %) patients had an age >35 year. Median age was 52 years (range 24–80) (Table 1).

**Table 1:** Age distribution

Age in years	No	%
<35	21	8.8
>35	217	91.2
Range	24–80	
Mean ± SD	52.0 ± 10.8	

Invasive ductal carcinoma represented 228 (95 %) of all patients which was considered the commonest histopathological type in this study. Grade II breast cancer patients were 182 (76.5 %) patients (Table 2).

**Table 2:** Histopathological types and grade

Histology	No	%
IDC	228	95
ILC	10	4.2
Grade		
I	8	3.4
II	182	76.5
III	48	20.2

Of the included 238 patients, 146 (61.3 %) patients presented with a clinical tumor of 2 cm or less.

One hundred twenty-one (50.8 %) patients had no pathological LNS.

Stage I–II breast cancer patients were 191 (80.3 %) patients. Forty-seven (19.7 %) patients were stage III breast cancer and underwent BCS. Thirteen (27.6 %) patients had neoadjuvant chemotherapy before surgery (Tables 3 and 4).

**Table 3:** Tumour characteristics

Tumour characteristics	No	%
Size		
T1	146	61.3
T2	86	36.1
T3	6	2.5
LNs		
N0	121	50.8
N1	77	32.4
N2	25	10.5
N3	15	6.3
Stage		
I	74	31.1
II	117	49.2
III	47	19.7

**Table 4:** Stage and procedure performed

Stage	No & %	Operation
I&II	191 (43 %)	BCS 191 (43 %)
III	47 (21.3 %)	BCS 47 (21.3 %) (13 patients after neoadjuvant chemotherapy)

Luminal A subtype represented 223 (93.7 %) of all patients. Triple negative subtype represented eight (3.3 %) of all patients. (Tables 5 and 6).

**Table 5:** Hormone receptors

Hormone receptors	No	%
Estrogen		
-ve	12	5
+	24	10.1
++	120	50.4
+++	82	34.5
Progesterone		
-ve	19	8
+	59	24.8
++	104	43.7
+++	56	23.5
Her2		
-ve	209	87.8
+	16	6.7
++	1	0.4
+++	12	5

**Table 6:** Biological subtypes

Type	No	%
Luminal A	223	93.7
Luminal B	4	1.7
Triple negative	8	3.3
Her2 enriched	3	1.3

Histopathological examination revealed that 47 (19.7 %) patients had excess intraductal component of the tumor, whereas 58 (24.4 %) had lympho-vascular invasion.

Adjuvant radiotherapy was given to 238 (100 %) patients.

Adjuvant chemotherapy was given to 201 (84.5 %) patients.

Hormonal therapy was given to 215 (90.3 %) patients (Table 7).

**Table 7:** Adjuvant treatment

Adjuvant treatment	Patients
Chemotherapy	No: 37 (15.5 %) Yes: 201 (84.5 %)
Radiotherapy	Yes: all cases
Hormonal	No: 23 (9.7 %) Yes: 215 (90.33 %)

After a median follow-up of 5 years (range 1–10 years), 16 (6.72 %) patients had loco-regional recurrence (LRR), whereas 10 (4.2 %) patients had distant recurrence. Using the Kaplan-Meier method to determine the loco-regional recurrence-free survival and distant recurrence-free survival; it was found to be 92.7 and 96.4 %, respectively.

### Prognostic factors for loco-regional recurrence

**Fig. 1:**
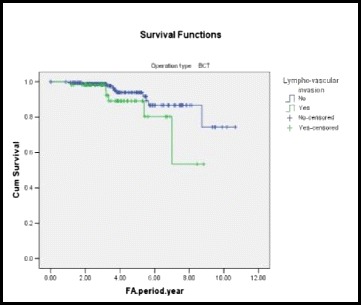
A Kaplan-Meier plot showing LRR-free survival by lympho-vascular invasion in BCS group

Analysis by the Cox proportional hazards model (Table 8), according to treatment actually given, demonstrated that relative risk of LRR for patients with lympho-vascular invasion compared with those without lympho-vascular invasion was 2.55 after BCS. The 5-year free survival of LRR after BCS was 89.1 % for patients with lymph-vascular invasion and 94 % for those without lympho-vascular invasion; (Fig. 1).

**Table 8:** Cox regression for loco-regional recurrence

Variables in the equation							
Operation type		B	SE	Sig.	HR	95.0 % CI for HR	
						Lower	Upper
BCT	Age	.003	.024	.901	1.003	.957	1.052
	T	.244	.680	.719	1.277	.337	4.839
	LNs	.900	.691	.193	2.461	.635	9.541
	Stage	−.911	.885	.303	.402	.071	2.277
	Grade	−1.322	.603	.028	.267	.082	.870
	IDC	.799	.596	.180	2.223	.691	7.151
	Invasion	.938	.613	.126	2.555	.768	8.497
	Postop_chemo	−1.137	.745	.127	.321	.074	1.382
	Horm	−1.858	.658	.005	.156	.043	.567

HR > 1 is considered as a risk factor

Patients with high nodal status have a 2.46 times higher risk of developing LRR after BCS compared with those with low nodal status.

**Fig. 2:**
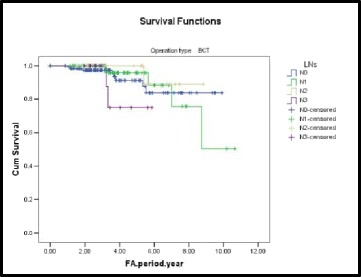
A Kaplan-Meier plot showing LRR-free survival by nodal status in BCS group

The 5-year free survival of LRR after BCS was 75 % for N3 patients and 95.7 % for N1 patients (Fig. 2).

**Fig. 3:**
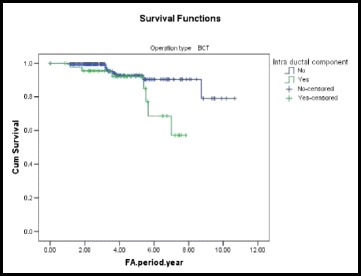
A Kaplan-Meier plot showing LRR-free survival by intraductal component in BCS group

The relative risk of LRR for patients with intraductal component was 2.22 times after BCS compared with those without intraductal component. The 5-year free survival of LRR after BCS was 92.1 % for patients with intraductal component and 92.8 % for patients without intraductal component (Fig. 3).

**Fig. 4:**
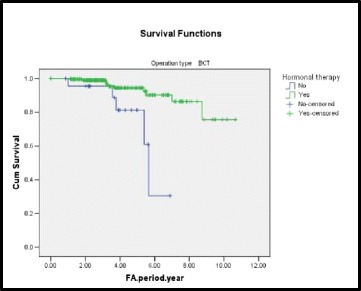
A Kaplan-Meier plot showing LRR-free survival by hormonal therapy in BCS group

The 5-year free survival of LRR after BCS was 94.4 % for patients who received hormonal therapy and 81.3 % for patients who did not receive hormonal therapy (Fig. 4).

### Prognostic factors for distant recurrence

According to the results of the multivariate Cox proportional hazards survival analysis, tumor size, nodal status, and histologic grade were all highly predictor factors of distant recurrence after BCS (Table 9).

**Table 9:** Cox regression for distal recurrence

Variables in the equation							
Operation type		B	SE	Sig.	HR	95.0 % CI for HR	
						Lower	Upper
BCT	Age	−.014	.036	.684	.986	.919	1.057
	T	.897	.785	.253	2.453	.527	11.417
	LNs	.656	.635	.302	1.927	.555	6.692
	Stage	.065	.947	.945	1.067	.167	6.825
	Grade	.392	.744	.598	1.480	.344	6.356
	IDC	.132	.776	.864	1.142	.250	5.221
	Invasion	−1.233	.949	.194	.291	.045	1.870
	Postop_XRT	−.293	1.214	.809	.746	.069	8.056
	Horm	−1.759	.848	.038	.172	.033	.907

HR > 1 is considered as a risk factor

**Fig. 5:**
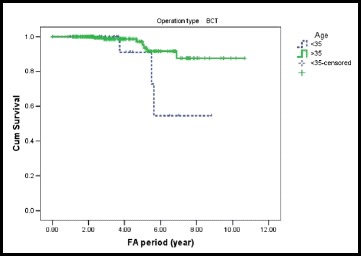
A Kaplan-Meier plot showing distant recurrence-free survival by age in BCS group

In addition, young age is an independent predictor of distant recurrence after BCS. The 5-year free survival of distant recurrence after BCS was 90.9 % for patient aged 35 years and younger and 97 % for patients over 35 years (Fig. 5).

**Fig. 6:**
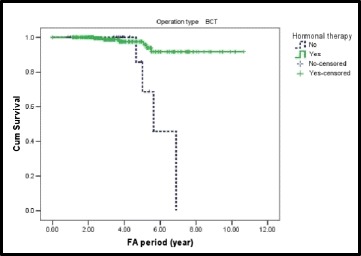
A Kaplan-Meier plot showing distant recurrence-free survival by hormonal therapy in BCS group

Hormonal therapy is also a protecting factor against distant recurrence after BCS with HR = 0.17; CI 95 % = 0.033–0.91. The 5-year survival of distant recurrence after BCS was 97.5 % for patients who received hormonal therapy and 85.7 % for those who did not receive hormonal therapy (Fig. 6).

## Discussion

The surgical treatment of breast cancer has been changed during the previous decades towards a less extensive surgery. Breast conservative surgery (BCS) is a model of this type of surgery which in properly selected patients provides local control of the disease. Based on the systemic disease concept of cancer breast, the removal of the primary does not obviate the risk of distant spread. Thus, our concern in the present study was to analyze the risk factors associated with loco-regional recurrence after BCS as local recurrence will obviate the purpose of breast conservation [12, 13].

The study revealed that the presence of an extensive intraductal component (EIC), lympho-vascular invasion and nodal status are associated with an increased risk of LRR after BCS.

EIC is an established risk factor for LRR after breast conservative therapy (BCT) [5, 14–16]. Invasive breast carcinoma is accompanied by an extensive component of DCTS in 15–30 % of patients.

DCIS grows along the ducts in the breast without invasion of the underlying tissue, which results in a non-palpable lesion difficult to remove with tumor-free margins. When EDCIs are completely removed with negative tumor margins, it loses its predicative value for LRR [5, 14–19]. In a pooled analysis of the EORTC 10801 and the DBCG 82 TM trials, lympho-vascular invasion causes a higher risk of LRR after BCT [20].

This result is also concordant with the observations made by Salim Alrahbi et al., Zahra MA Mohammed et al., and Bent Ejlertsen et al. [21–23].

In agreement with others, positive nodal status is a predictor for LRR in patients of BCT group [24]. Our study suggests a favorable effect of postoperative radiotherapy and adjuvant systemic treatment on LRR and distant metastases for BCS [12, 13, 20]. Today, the widespread use of adjuvant systemic therapy (chemotherapy and endocrine therapy) for both node-positive and node-negative breast cancer, coupled with improvements in the mammographic and pathological assessment of patients undergoing breast-conserving surgery, has resulted in decreased incidence of local failure [25, 26].

A study in Japan investigating the occurrence of ipsilateral breast recurrence, after long-term follow-up of patients with early breast cancer, after breast-conservative surgery, found it to be significantly associated with young age, positive surgical margin, and omission of radiation therapy [27].

Our study reveals that tumor size, nodal status, and high histologic grade are predictors for distant recurrence after BCS. Various studies have described an increased rate of distant metastases among those patients [14, 20, 28–33].

Patients 35 years of age or younger appeared to have an increased risk of distant disease. The increased risk was much more prominent after BCT.

The adverse effect of young age on prognosis has been noted in several other studies and suggests that breast cancer in younger women is biologically more aggressive disease, possibly requiring more aggressive initial treatment. The question arises whether LRR might be a source of distant spread in some patients in the youngest age group. Unfortunately, numbers in the current study did not allow us to find the answer to this question [17, 34, 35].

## Conclusions

Risk factors for loco-regional recurrence and distant metastases play an important role in the decision for the treatment of breast cancer. This decision-making can be optimized if patients at high risk for loco-regional recurrence can be identified.

- Lympho-vascular invasion, EIC, and high nodal status are risk factors for LRR after BCT.
- Tumor size, nodal status, high histologic grade, and breast cancer diagnosed at a young age (<35 years) are correlated with higher distant recurrence rates after BCT.
- Radiotherapy and hormonal therapy have a great role in decreasing the development of recurrence after BCS.
